# Modified Unruh effect from generalized uncertainty principle

**DOI:** 10.1140/epjc/s10052-018-6209-y

**Published:** 2018-09-10

**Authors:** Fabio Scardigli, Massimo Blasone, Gaetano Luciano, Roberto Casadio

**Affiliations:** 1 Dipartimento di Matematica, Politecnico di Milano, Piazza Leonardo da Vinci 32, 20133 Milan, Italy; 2 Institute-Lorentz for Theoretical Physics, Leiden University, P.O. Box 9506, Leiden, The Netherlands; 3 Dipartimento di Fisica, Università di Salerno, 84084 Fisciano, SA Italy; 4 I.N.F.N., Gruppo Collegato di Salerno, Salerno, Italy; 5 Dipartimento di Fisica e Astronomia, Alma Mater Università di Bologna, via Irnerio 46, 40126 Bologna, Italy; 6 I.N.F.N., Sezione di Bologna, viale Bert Pichat 6/2, 40127 Bologna, Italy

## Abstract

We consider a generalized uncertainty principle (GUP) corresponding to a deformation of the fundamental commutator obtained by adding a term quadratic in the momentum. From this GUP, we compute corrections to the Unruh effect and related Unruh temperature, by first following a heuristic derivation, and then a more standard field theoretic calculation. In the limit of small deformations, we recover the thermal character of the Unruh radiation. Corrections to the temperature at first order in the deforming parameter are compared for the two approaches, and found to be in agreement as for the dependence on the cubic power of the acceleration of the reference frame. The dependence of the shifted temperature on the frequency is also pointed out and discussed.

## Introduction

In the last 30 years, many studies have converged on the idea that the Heisenberg uncertainty principle (HUP) [1] should be modified when gravitation is taken into account. In microphysics, gravity is usually neglected on the ground of its weakness, when compared with the other fundamental interactions. However, this argument should not apply when one wants to address fundamental questions in Nature. In this perspective, gravity should be included, especially when we discuss the formulation of fundamental principles like Heisenberg’s principle. And in fact, gravitation has always played a pivotal role in the generalization of the HUP, from the early attempts [2–5], to the more recent proposals, like those in string theory, loop quantum gravity, deformed special relativity, non-commutative geometry, and studies of black hole physics [6–18].

A possible way for this generalization is to reconsider the well-known classical argument of the Heisenberg microscope [1]. The size $\delta x$ of the smallest detail of an object, theoretically detectable with a beam of photons of energy *E*, is roughly given by (if we assume the dispersion relation $E=p$)^1^

1 $$\begin{aligned} \delta x \simeq \frac{\hbar }{2\, E}, \end{aligned}$$

so that increasingly large energies are required to explore decreasingly small details. In its original formulation, Heisenberg’s gedanken experiment ignores gravity. Nevertheless, gedanken experiments involving formation of gravitational instabilities in high energy scatterings of strings [6–9], or gedanken experiments taking into account the possible formation, in high energy scatterings, of micro black holes with a gravitational radius $R_S=R_S(E)$ proportional to the (centre-of-mass) scattering energy *E* (see Ref. [15]), suggest that the usual uncertainty relation should be modified as

2 $$\begin{aligned} \delta x \simeq \frac{\hbar }{2\, E} + \beta \, R_S(E), \end{aligned}$$

where $\beta$ is a dimensionless parameter. Recalling that $R_S\simeq 2\,G_{\mathrm{N}}\, E = 2\, \ell _{\mathrm{p}}^2\, E/\hbar$, we can write

3 $$\begin{aligned} \delta x \simeq \frac{\hbar }{2\, E} + 2\beta \, \ell _{\mathrm{p}}^2\frac{E}{\hbar } = \ell _{\mathrm{p}}\left( \frac{m_{\mathrm{p}}}{E} + \beta \, \frac{E}{m_{\mathrm{p}}} \right) . \end{aligned}$$

This kind of modification of the uncertainty principle was also proposed in Ref. [16].

The dimensionless deforming parameter $\beta$ is not (in principle) fixed by the theory, although it is generally assumed to be of order one. This happens, in particular, in some models of string theory (see again for instance Refs. [6–9]), and has been confirmed by an explicit calculation in Ref. [19]. However, many studies have appeared in literature, with the aim to set bounds on $\beta$ (see, for instance, Refs. [20–24]).

The relation (3) can be recast in the form of an uncertainty relation, namely a deformation of the standard HUP, usually referred to as Generalized Uncertainty Principle (GUP),

4 $$\begin{aligned} \varDelta x\, \varDelta p \ge \frac{\hbar }{2} \left[ 1 +\beta \left( \frac{\varDelta p}{m_{\mathrm{p}}}\right) ^2 \right] . \end{aligned}$$

For mirror-symmetric states (with $\langle {\hat{p}} \rangle = 0$), the inequality (4) is equivalent to the commutator

5 $$\begin{aligned} \left[ {\hat{x}},{\hat{p}}\right] = i \hbar \left[ 1 +\beta \left( \frac{{\hat{p}}}{m_{\mathrm{p}}} \right) ^2 \right] , \end{aligned}$$

since $\varDelta x\, \varDelta p \ge (1/2)\left| \langle [{\hat{x}},{\hat{p}}] \rangle \right|$. Vice-versa, the commutator (5) implies the inequality (4) for any state. The GUP is widely studied in the context of quantum mechanics [25–27], quantum field theory [28], quantum gravity [29, 30], and for various deformations of the quantization rules [29–31].

The above $\beta$-deformed commutator (5) will be the starting point of the present investigation. In what follows, using (5), we shall describe the Unruh effect (known also as Fulling–Davies–Unruh effect [32–34]), thereby calculating corrections to the Unruh temperature to first order in $\beta$. A direct derivation of the Unruh effect from the HUP has been given in Ref. [35]. On the other hand, the necessity of this effect for the internal consistency of QFT has been confirmed by arguments based both on general covariance [36–39] and thermodynamic [40]. Moreover, non-trivial modifications to the Unruh spectrum have been pointed out also, in the GUP context, in Refs. [41–44], and in different contexts in Refs. [45, 46]. For instance, it has been shown that flavor mixing does spoil the thermal character of the Unruh radiation [47, 48], thus opening new stimulating scenarios.

## Heuristic derivation of Unruh effect from uncertainty relations

In this section we derive the Unruh temperature [34] starting directly from the HUP. Simple classical physics relations will be used together with the quantum principle, following closely Ref. [35] (see also the recent Ref. [49]). This procedure will then allow us to estimate what kind of corrections are induced by a GUP.

Let us consider some elementary particles, for example electrons, kept at rest in an uniformly accelerated frame. The kinetic energy acquired by each of these particles while the accelerated frame moves a distance $\delta x$ will be given by

6 $$\begin{aligned} E_k = m\,a\,\delta x, \end{aligned}$$

where *m* is the mass of the particle and *a* the acceleration of the frame, and therefore of the particle. Now, suppose this energy is sufficient to create *N* pairs of the same kind of particles from the quantum vacuum. Namely, we set

7 $$\begin{aligned} E_k \simeq 2\,N\, m, \end{aligned}$$

and find that the distance along which each particle must be accelerated in order to create *N* pairs is

8 $$\begin{aligned} \delta x \simeq 2\,\frac{N}{a}. \end{aligned}$$

The original particles and the pairs created in this way are localized inside a spatial region of width $\delta x$, therefore the fluctuation in energy of each single particle is

9 $$\begin{aligned} \delta E \simeq \frac{\hbar }{2\, \delta x} \simeq \frac{\hbar \, a}{4\, N}. \end{aligned}$$

If we interpret this fluctuation as a classical thermal agitation of the particles, we can write

10 $$\begin{aligned} \frac{3}{2}\,k_{\mathrm{B}}\,T \simeq \delta E \simeq \frac{\hbar \, a}{4\,N}, \end{aligned}$$

or

11 $$\begin{aligned} T = \frac{\hbar \, a}{6\,N\,k_{\mathrm{B}}}. \end{aligned}$$

On comparing with the well-known Unruh temperature [34],

12 $$\begin{aligned} T_{\mathrm{U}} = \frac{\hbar \, a}{2\,\pi \, k_{\mathrm{B}}}, \end{aligned}$$

we can set the arbitrary parameter *N* and obtain an effective number of pairs $N=\pi /3\simeq 1$.

Now we repeat the same argument using the GUP. Upon replacing Eq. (8) into Eq. (3), and interpreting the energy fluctuation $\delta E$ in terms of a classical thermal bath, we find

13 $$\begin{aligned} 2\,\frac{N}{a} \simeq \frac{\hbar }{3\, k_{\mathrm{B}}\, T} + \beta \, \ell _{\mathrm{p}}^2\, \frac{3\, k_{\mathrm{B}}\, T}{\hbar }. \end{aligned}$$

Requiring that the *T* equals the Unruh temperature (12) for $\beta \rightarrow 0$ again fixes $N=\pi /3\simeq 1$, and we finally obtain

14 $$\begin{aligned} \frac{2\,\pi }{a} \simeq \frac{\hbar }{k_{\mathrm{B}}\, T} + 9\beta \, \ell _{\mathrm{p}}^2\, \frac{k_{\mathrm{B}}\, T}{\hbar } = \ell _{\mathrm{p}}\left( \frac{2m_{\mathrm{p}}}{k_{\mathrm{B}}\,T} + 9\,\beta \,\frac{k_{\mathrm{B}}\, T}{2m_{\mathrm{p}}} \right) . \end{aligned}$$

This relation can be easily inverted for $T=T(a)$. However, it is reasonable to assume that $\beta \,k_{\mathrm{B}} T/m_{\mathrm{p}}\sim \beta \,m/m_{\mathrm{p}}$ is very small for any fundamental particle with $m\ll m_{\mathrm{p}}$. We can therefore expand in $\beta \,m/m_{\mathrm{p}}$ and find

15 $$\begin{aligned} T \simeq T_{\mathrm{U}} \left( 1 + \frac{9\,\beta }{4}\,\frac{\ell _{\mathrm{p}}^2\,a^2}{\pi ^2} \right) = T_{\mathrm{U}} \left[ 1 + \frac{9\,\beta }{4} \left( \frac{k_{\mathrm{B}}\,T_{\mathrm{U}}}{m_{\mathrm{p}}} \right) ^2 \right] . \end{aligned}$$

We also notice an interesting physical property suggested by Eq. (14), that is, by the GUP. In order to maintain the inverted relation $T=T(a)$ physically meaningful (i.e. the temperature must be a real number), there will be a maximal value for the acceleration, namely

16 $$\begin{aligned} a \lesssim \frac{\pi }{3 \, \sqrt{\beta }\, \ell _{\mathrm{p}}}, \end{aligned}$$

and a corresponding maximal value for the Unruh–Davies temperature,

17 $$\begin{aligned} k_{\mathrm{B}}\,T_{\mathrm{U}} \lesssim \frac{m_{\mathrm{p}}}{3\,\sqrt{\beta }}. \end{aligned}$$

These ideas and estimates naturally make contact with those reported, for example, in Refs. [50, 51].

## Quantization of a massive scalar field in accelerated frames

In this section we briefly review the quantization of a massive scalar field for an accelerated observer. This will serve as a basis for the analysis of Sect. 4, where the deformation of the algebra discussed above is implemented. For the sake of simplicity, we will work in $1+1$-dimensions, using the Minkowski metric with the conventional signature $ds^2= \eta _{\mu \nu }\,dx^\mu \,dx^\nu =dt^2-dx^2$. In this section we set $\hbar =c=k_{\mathrm{B}}=1$, unless otherwise explicitly stated.

### Minkowski space-time

For an inertial observer, the scalar field in the usual plane-wave representation reads

18 $$\begin{aligned} \phi (\mathbf{x}) = \int dk\, \left[ a_{k}\,U_{k}(\mathbf{x}) + a_{k}^\dagger \,U_{k}^{*}(\mathbf{x}) \right] , \end{aligned}$$

where $\mathbf{x}\equiv \{t, x\}$ denotes the set of Minkowski coordinates. The positive frequency plane-waves of momentum *k* are given by

19 $$\begin{aligned} U_{k}(\mathbf{x}) = {\left( 4\,\pi \,\omega _{k}\right) }^{-\frac{1}{2}}\, e^{i\left( k\,x-\omega _{k}\, t\right) }, \end{aligned}$$

where $\omega _{k}=\sqrt{m^2+k^2}$, *m* being the mass of the field. Within the framework of canonical quantum field theory (QFT), the annihilation and creation operators for Minkowski quanta, to wit $a_{k}$ and $a^\dagger _{k}$, satisfy the standard commutation relation

20 $$\begin{aligned} \left[ a_k, a_{k'}^\dagger \right] = \delta (k-k'), \end{aligned}$$

with all other commutators vanishing. The ordinary Minkowski vacuum is accordingly defined by $a_k\,|0_M\rangle =0$ for all modes *k*.

As a tool for extending this quantization scheme to an accelerated observer, let us now introduce the less familiar Lorentz-boost eigenfunctions [47]. Boost modes are related to the plane-waves in Eq. (19) by

21 $$\begin{aligned} {\widetilde{U}}_{\varOmega }^{(\sigma )}(\mathbf{x}) = \int dk\, p_\varOmega ^{(\sigma )*}(k)\,U_{k}(\mathbf{x}), \end{aligned}$$

where

22 $$\begin{aligned} p_\varOmega ^{(\sigma )}(k) = \frac{1}{\sqrt{2\,\pi \,\omega _{k}}}\, \left( \frac{\omega _{k}+k}{\omega _{k}-k}\right) ^{i\,\sigma \,\varOmega /2}, \end{aligned}$$

and $\sigma =\pm ,\,\, 0<\varOmega <\infty .$ The physical meaning of the quantum numbers $\varOmega$ and $\sigma$ will be discussed in the next section. In terms of the modes (21), the spectral representation of the field operator can be written as^2^

23 $$\begin{aligned} \phi (\mathbf{x}) = \int _{0}^{+\infty } d\varOmega \, \sum _{\sigma } \left[ d_{\varOmega }^{(\sigma )}\,{\widetilde{U}}_{\varOmega }^{(\sigma )}(\mathbf{x}) + d_{\varOmega }^{(\sigma )\dagger }\,{\widetilde{U}}_{\varOmega }^{(\sigma )*}(\mathbf{x}) \right] . \end{aligned}$$

It is easy to prove that the two quantum constructions introduced above are equivalent to each other. For this purpose, let us equate the field-expansions (18) and (23) on a space-like hypersurface. By using Eq. (21), it follows that

24 $$\begin{aligned} d_{\varOmega }^{(\sigma )} = \int dk\, p_\varOmega ^{(\sigma )}(k)\,a_{k}. \end{aligned}$$

Since the operators $d_{\varOmega }^{\,(\sigma )}$ are linear combinations of the Minkowski annihilators $a_{k}$ alone, they also annihilate the Minkowski vacuum $|0_M\rangle$. Moreover, by exploiting the completeness and orthonormality of the set of functions $\left\{ p_\varOmega ^{(\sigma )}\right\}$ (see Ref. [54]), it can be shown that the transformation (24) is canonical, so that

25 $$\begin{aligned} \left[ d_{\varOmega }^{(\sigma )},d_{\varOmega '}^{\,(\sigma ')\dagger }\right] = \delta _{\sigma \sigma '}\,\delta (\varOmega -\varOmega '), \end{aligned}$$

with all other commutators vanishing. Equations (24) and (25) allow us to interpret also the $d_{\varOmega }^{\,(\sigma )}$ as annihilation operators of Minkowski quanta. This implies that the field-expansions Eqs. (18) and (23) can be used equivalently within the framework of canonical quantization in Minkowski space-time. For our purposes, in what follows it will be convenient to employ the latter.

### Rindler space-time

The foregoing discussion applies to inertial observers in Minkowski space-time. In order to investigate GUP effects on the Unruh radiation [34], let us now review the Rindler–Fulling field-quantization in a uniformly accelerating frame [32]. By introducing the usual Rindler coordinates $\{\eta ,\xi \}$, in place of $\{t, x\}$, we have

26 $$\begin{aligned} t = \xi \,\sinh \eta , \quad x= \xi \,\cosh \eta , \qquad -\infty<\eta ,\ \xi <\infty , \end{aligned}$$

and the Minkowski line element takes the well-known form

27 $$\begin{aligned} ds^2 = dt^2-dx^2 = \xi ^2\,d\eta ^2 -d\xi ^2. \end{aligned}$$

**Fig. 1:**
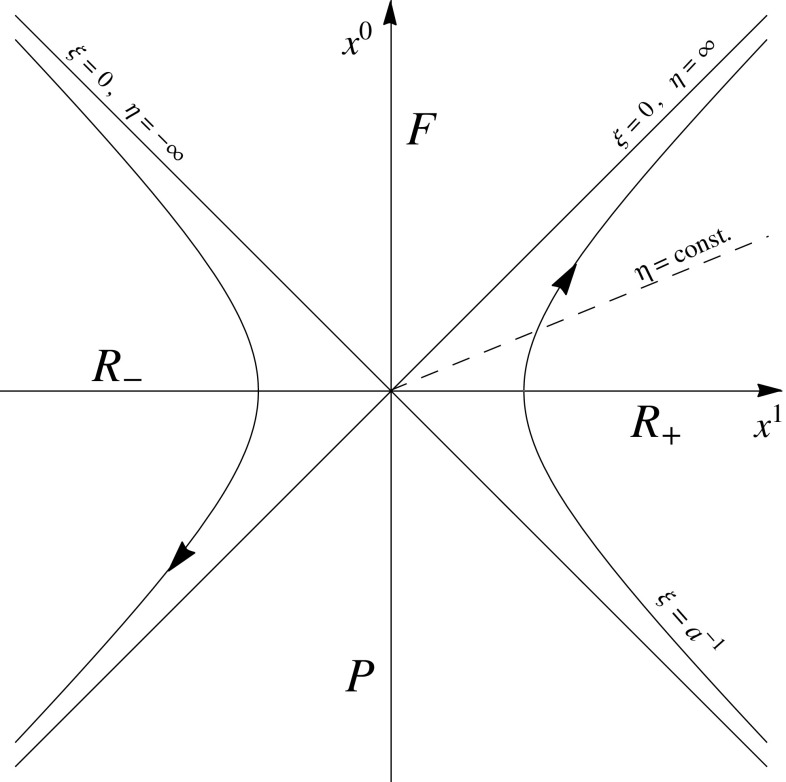
The proper coordinate system of a uniformly accelerated observer in the Minkowski spacetime. The branch of hyperbola $\xi =a^{-1}$ represents the worldline of an observer with proper acceleration *a*

As $\xi$ and $\eta$ range from $-\infty$ to $\infty$, the Rindler coordinates cover only two sections of Minkowski space-time, specifically the right wedge $R_+=\left\{ \mathbf{x}\,|\,x>|t|\right\}$ for $\xi >0$, and the left wedge $R_-=\left\{ \mathbf{x}\,|\,x<-|t|\right\}$ for $\xi <0$ (see Fig. 1). Since the components of the metric in these coordinates do not depend on $\eta$, Eq. (27) describes a static spacetime with Killing vector $\mathbf{B}=\partial _\eta$.

The worldline of a uniformly accelerated (Rindler) observer with proper acceleration |*a*| is given by

28 $$\begin{aligned} \xi (\tau ) = \mathrm {const} \equiv a^{-1}, \end{aligned}$$

where $\tau =\eta /a$ is the proper time along the accelerated trajectory (restoring $c\ne 1$, one has $\xi (\tau )=c^2/a$, and $\tau =\eta c/a$; that is, $\eta =a\tau /c$ is dimensionless). This is a branch of hyperbola in the (*t*, *x*) plane, whose null asymptotes $t=\pm x$ act respectively as future and past event horizons for the Rindler observer.

Because of the non-trivial structure of Rindler space-time, the wedges $R_\pm$ are causally disconnected from each other [53]. The positive frequency solutions of the Klein–Gordon equation in Rindler coordinates thus take the form^3^

29 $$\begin{aligned} u_\varOmega ^{(\sigma )}(\mathbf{x}) = N_\varOmega \, \theta (\sigma \,\xi )\, K_{i\,\varOmega }^{(\sigma )}(m\,\xi )\, e^{-i\,\sigma \,\varOmega \,\eta }, \end{aligned}$$

where $\varOmega$ is the Rindler frequency with respect to the time $\eta$,^4^ $\sigma =\pm$ refers to the right/left wedges $R_\pm$ and $K_{i\,\varOmega }$ is the modified Bessel function of the second kind. In this context, we do not need to specify the normalization factor $N_\varOmega$ (for more details, see Ref. [54]). Furthermore, the Heaviside function $\theta (\sigma \,\xi )$ was inserted into Eq. (29) in order to constrain the Rindler modes to only one of the two disconnected wedges $R_\pm$.

Using Eq. (29), we can now expand the scalar field operator in the Rindler space-time as follows

30 $$\begin{aligned} \phi (\mathbf{x}) = \int _{0}^{+\infty } d\varOmega \, \sum _\sigma \, \left[ b^{(\sigma )}_{\varOmega }\,u_{\varOmega }^{(\sigma )}(\mathbf{x}) +b^{(\sigma )\dagger }_{\varOmega }\,u_{\varOmega }^{(\sigma )*}(\mathbf{x}) \right] , \end{aligned}$$

where the ladder operators $b^{\,(\sigma )}_\varOmega$ and ${b^{\,(\sigma )\dagger }_{\varOmega }}$ are assumed to satisfy the canonical commutation relations

31 $$\begin{aligned} \left[ b_\varOmega ^{(\sigma )}, b_{\varOmega '}^{( \sigma ')\dagger }\right] = \delta _{\sigma \sigma '}\, \delta (\varOmega -\varOmega '), \end{aligned}$$

with all other commutators vanishing. The Rindler vacuum is accordingly defined by $b_\varOmega ^{(\sigma )}|0_{\mathrm{R}}\rangle = 0$, for all values of $\sigma$ and $\varOmega$.

The connection between the two quantization schemes, for inertial and accelerated observers, can now be investigated in detail. Specifically, we compare the field expansions (23) and (30) on a spacelike hypersurface $\varSigma$ lying in the Rindler manifold $R_\pm$. A straightforward calculation leads to the following Bogoliubov transformation [54]

32 $$\begin{aligned} b^{(\sigma )}_{\varOmega } = \left[ 1+\mathcal {N}(\varOmega )\right] ^{1/2}\, d_{\varOmega }^{(\sigma )} + \mathcal {N}(\varOmega )^{1/2}\,d_{\varOmega }^{(-\sigma )\dagger }, \end{aligned}$$

where

33 $$\begin{aligned} \mathcal {N}(\varOmega ) = \frac{1}{e^{2\,\pi \,\varOmega }-1} \end{aligned}$$

is the Bose–Einstein distribution. Using Eq. (32), we can now calculate the spectrum of Rindler quanta in the Minkowski vacuum $|0_{\mathrm{M}}\rangle$,

34 $$\begin{aligned} \langle 0_{\mathrm{M}}|\,b_{\varOmega }^{(\sigma )\dagger }\,b_{\varOmega '}^{ (\sigma ')}\,|0_{\mathrm{M}}\rangle = \mathcal {N}(\varOmega )\,\delta _{\sigma \sigma '}\,\delta (\varOmega -\varOmega '). \end{aligned}$$

It then follows that a uniformly accelerated observer perceives the Minkowski vacuum as a thermal bath of Rindler quanta with a temperature proportional to the acceleration (the Unruh effect [34]). Restoring our standard units ($c=1$, $\hbar \ne 1$, $k_{\mathrm{B}}\ne 1$), we can in fact write

35 $$\begin{aligned} 2\,\pi \,\varOmega = \frac{2\pi }{a}\,a\,\varOmega = \frac{\hbar \,a\, \varOmega }{k_{\mathrm{B}}\,T_{\mathrm{U}}} = \frac{\hbar \,\omega }{k_{\mathrm{B}}\,T_{\mathrm{U}}}, \end{aligned}$$

where $\omega =a\,\varOmega$ is the frequency measured by the Rindler observer and $T_{\mathrm{U}}$ the Unruh temperature (12).

## GUP and modified Unruh temperature

In the previous section, the Unruh temperature (12) has just been re-derived within the framework of canonical QFT. At this stage, one may wonder how such a result gets modified when starting from the GUP commutator in Eq. (5). To answer this question, an intermediate step concerning the effects of GUP on a quantum one-dimensional harmonic oscillator turns out to be useful. In this context, we note that the ladder operators *A* and $A^\dagger$ of the deformed algebra for the one-dimensional harmonic oscillator are linked to ${\hat{x}}={\hat{x}}^\dagger$ and ${\hat{p}}={\hat{p}}^\dagger$ by the usual relations

36 $$\begin{aligned} A= & {} \displaystyle \frac{1}{\sqrt{2\,m\,\hbar \,\omega }} \left( m\omega {\hat{x}} + i \,{\hat{p}}\right) \nonumber \\ A^{\dagger }= & {} \displaystyle \frac{1}{\sqrt{2\,m\,\hbar \,\omega }} \left( m\omega {\hat{x}} - i\, {\hat{p}}\right) , \end{aligned}$$

and their inverse relations

37 $$\begin{aligned} \begin{array}{l} {\hat{x}} = \displaystyle \sqrt{\frac{\hbar }{2\,m\,\omega }} (A^\dagger + A)\\ {\hat{p}} = \displaystyle i\,\sqrt{\frac{m\,\hbar \,\omega }{2}} (A^\dagger - A). \end{array} \end{aligned}$$

It is then easy to see that

38 $$\begin{aligned} {[}A,A^\dagger ]\,=\,\frac{1}{i\hbar }\,[{{\hat{x}}}, {{\hat{p}}}] \end{aligned}$$

and, due to the modified commutator (5) between ${{\hat{x}}}$ and ${{\hat{p}}}$, the deformed algebra for the one-dimensional harmonic oscillator should be written as

39 $$\begin{aligned}{}[A, A^\dagger ] = \frac{1}{1-\alpha } [1 -\alpha (A^\dagger \, A^\dagger +A\,A -2\,A^\dagger \, A ) ], \end{aligned}$$

where

40 $$\begin{aligned} \alpha =\beta \,\frac{m\,\hbar \, \omega }{2\,m_{\mathrm{p}}^2}, \end{aligned}$$

with *m* and $\omega$ being the mass and frequency of the harmonic oscillator, respectively. The modified quantization rules (39) can be now extended in a natural way to a scalar field in the plane-wave representation, if we consider that, for a given momentum *k*, the energy $\hbar \,\omega _k$ of the scalar field plays the role of the mass *m* of the harmonic oscillator. The deformation parameter $\alpha$ can be then suitably redefined as

41 $$\begin{aligned} \tilde{\alpha }= \beta \,\frac{\hbar ^2 \omega _k^2}{2\,m_{\mathrm{p}}^2}= 2\,\beta \,\ell _{\mathrm{p}}^2\,\omega _k^2 \end{aligned}$$

and the commutator between ladder operators becomes

42 $$\begin{aligned}{}[A_k, A_{k'}^\dagger ]= & {} \frac{1}{1-\tilde{\alpha }} [1 - \tilde{\alpha } (A_{k}^\dagger \, A_{k'}^\dagger \nonumber \\&+\,A_{k}\, A_{k'} -2\,A_{k}^\dagger \, A_{k'} ) ] \delta (k-k'). \end{aligned}$$

In Sect. 3, we have seen that the scalar field for an inertial observer can be quantized both using plane-waves and boost-modes (see Eqs. (18) and (23), respectively). In that context, the choice between these two representations is just a matter of convenience, since the corresponding sets of ladder operators $a_k$ and $d_{\varOmega }^{(\sigma )}$ are related by the canonical transformation (24). With deformed quantization rules, however, Lorentz invariance is violated and such an equivalence is not guaranteed. Nevertheless, in the limit of very small deformation (that is, $\beta \, p^2\ll m_{\mathrm{p}}^2$), it appears reasonable to assume the same structure of the modified algebra for the two sets of operators. According to this argument, we thus conjecture the following deformation for the commutator in the boost-mode representation

43 $$\begin{aligned}&\left[ D_{\varOmega }^{(\sigma )}\,D_{\varOmega '}^{(\sigma ')\dagger }\right] \nonumber \\&\quad = \frac{1}{1-\gamma }\left[ 1-\gamma \,\left( D_{\varOmega }^{(\sigma )\dagger }\,D_{\varOmega '}^{(-\sigma ')\dagger } + D_{\varOmega }^{(\sigma )}\,D_{\varOmega '}^{(-\sigma ')} \right. \right. \nonumber \\&\qquad \left. \left. -D_{\varOmega }^{(\sigma )\dagger }\,D_{\varOmega '}^{(\sigma ')} -D_{\varOmega }^{(-\sigma )\dagger }\,D_{\varOmega '}^{(-\sigma ')} \right) \right] \delta _{\sigma \sigma '}\, \delta (\varOmega -\varOmega '), \qquad \end{aligned}$$

where $D_{\varOmega }^{\,(\sigma )}$ and $D_{\varOmega }^{\,(\sigma )\dagger }$ are the ladder operators in the deformed algebra and the deforming parameter $\gamma$ is defined by

44 $$\begin{aligned} \gamma = \beta \, \frac{\hbar ^2 \omega ^2}{2\,m_{\mathrm{p}}^2} = \beta \, \frac{\hbar ^2 a^2\,\varOmega ^2}{2\,m_{\mathrm{p}}^2} = 2\,\beta \,\ell _{\mathrm{p}}^2\,a^2\,\varOmega ^2, \end{aligned}$$

being $\omega =a\varOmega$ the Rindler frequency.

Some comments about Eq. (43) are needed. First, in order to adapt the deformed commutator (42) to the boost operators *D*, we have modified *ad hoc* the definition of the deforming parameter $\tilde{\alpha }$ by replacing the plane-frequency $\omega _k$ with the boost-mode frequency $\omega =a\,\varOmega$ [see Eq. (44)]. Furthermore, the commutator (43) has been multiplied by $\delta _{\sigma \sigma '}$ to ensure that the ladder operators in the right wedge $R_+$ are still commuting with the corresponding operators in the left wedge $R_-$. In addition, we symmetrized it with respect to $\sigma$ and $-\sigma$, so that

45 $$\begin{aligned} \left[ D_{\varOmega }^{(\sigma )},D_{\varOmega '}^{(\sigma ')\dagger }\right] = \left[ D_{\varOmega }^{(-\sigma )},D_{\varOmega '}^{(-\sigma ')\dagger }\right] . \end{aligned}$$

By exploiting this property and recasting the Bogoliubov transformation (32) in the form

46 $$\begin{aligned} B_{\varOmega }^{(\sigma )} = {\left[ 1+\mathcal {N}(\varOmega )\right] }^{1/2} \,D_{\varOmega }^{(\sigma )} + {\mathcal {N}(\varOmega )}^{1/2}\, D_{\varOmega }^{(-\sigma )\dagger }, \end{aligned}$$

one can verify that the deformation (43) induces an identical modification to the algebra of the Rindler operators *B*.

GUP effects on the Unruh temperature can now be investigated by calculating the distribution of *B*-quanta in the Minkowski vacuum $|0_{\mathrm{M}}\rangle$. By use of the transformation (46), it can be shown that

47 $$\begin{aligned} \langle 0_{\mathrm{M}}|\, B_{\varOmega }^{(\sigma )\dagger }\,B_{\varOmega '}^{ (\sigma ')}\, |0_{\mathrm{M}}\rangle = \frac{1}{\left( e^{2\pi \varOmega }-1\right) \left( 1-\gamma \right) }\, \delta _{\sigma \sigma '}\, \delta (\varOmega -\varOmega '),\nonumber \\ \end{aligned}$$

to be compared with the standard Bose–Einstein distribution in Eq. (34). As expected, the Unruh spectrum gets non-trivially modified by the deformed algebra (43) and loses its characteristic thermal behavior. However, for Rindler frequencies $\varOmega$ such that $\gamma \ll 1$, namely (since $\beta \sim 1$) for $\hbar \,\omega \ll m_{\mathrm{p}}$, we have $e^{-\gamma }\simeq 1-\gamma$, and Eq. (47) can be approximated as

48 $$\begin{aligned} \langle 0_{\mathrm{M}}|\, B_{\varOmega }^{(\sigma )\dagger }\,B_{\varOmega '}^{ (\sigma ')}\, |0_{\mathrm{M}}\rangle \simeq \frac{1}{e^{2\pi \varOmega -\gamma }-1}\, \delta _{\sigma \sigma '}\, \delta (\varOmega -\varOmega '), \end{aligned}$$

where we neglected the term linear in $\gamma$ in the denominator of the r.h.s. We can interpret Eq. (48) as a shifted Bose–Einstein thermal distribution by introducing a shifted Unruh temperature *T* such that the term $(2\pi \varOmega -\gamma )$ can be rewritten as

49 $$\begin{aligned} 2\pi \varOmega -\gamma \ = \ \frac{\hbar \,a\, \varOmega }{k_{\mathrm{B}}\,T_{\mathrm{U}}} - \gamma \ \equiv \ \frac{\hbar \,a\, \varOmega }{k_{\mathrm{B}}\,T}. \end{aligned}$$

We thus find for the shifted Unruh temperature

50 $$\begin{aligned} T= & {} \frac{T_{\mathrm{U}}}{1-\beta \,\pi \,\varOmega \,k_{\mathrm{B}}^2\,T_{\mathrm{U}}^2/m_{\mathrm{p}}^2} \nonumber \\\simeq & {} T_{\mathrm{U}} \left[ 1 + \beta \, \pi \, \varOmega \left( \frac{k_{\mathrm{B}}T_{\mathrm{U}}}{m_{\mathrm{p}}}\right) ^2 \right] \nonumber \\= & {} T_{\mathrm{U}} \left( 1+\beta \,\pi \,\varOmega \,\frac{\ell _{\mathrm{p}}^2\,a^2}{\pi ^2}\right) . \end{aligned}$$

We notice that such a modified temperature *T* contains an explicit dependence on the Rindler frequency $\varOmega$. This is due to the deformed structure of the commutator (5), which explicitly depends on ${\hat{p}}^2$, that is, essentially, on the energy of the considered quantum mode. So, it is not surprising to recover such an explicit dependence in the final formulae. Nevertheless, a simple thermodynamic argument allows us to get rid of this $\varOmega$-dependance. In fact, for small deformations, we are still close to the thermal black body spectrum. Therefore the vast majority of the Unruh quanta will be emitted around a Rindler frequency $\omega$ such that $\hbar \,\omega \simeq k_{\mathrm{B}}\,T_{\mathrm{U}}$, which means $\varOmega \approx 1/(2\pi )$. For this typical frequency, Eq. (50) reproduces quite closely the heuristic estimate (15). In fact

51 $$\begin{aligned} T \simeq T_{\mathrm{U}} \left[ 1 + \frac{\beta }{2} \left( \frac{k_{\mathrm{B}}T_{\mathrm{U}}}{m_{\mathrm{p}}}\right) ^2 \right] = T_{\mathrm{U}} \left( 1 + \frac{\beta }{2}\frac{\ell _{\mathrm{p}}^2\,a^2}{\pi ^2}\right) . \end{aligned}$$

Moreover, we can notice that, if we repeat the heuristic derivation of Sect. 2 in a $(1+1)$-dimensional framework (like the QFT calculation has been performed in Sect. 3), then in place of Eq. (10) we should write $k_{\mathrm{B}}T/2 \simeq \delta E \simeq \hbar a /4N$. This would yield a correction of a factor 9 in Eq. (15), which would now read

52 $$\begin{aligned} T \simeq T_{\mathrm{U}} \left( 1 + \frac{\beta }{4}\,\frac{\ell _{\mathrm{p}}^2\,a^2}{\pi ^2} \right) = T_{\mathrm{U}} \left[ 1 + \frac{\beta }{4} \left( \frac{k_{\mathrm{B}}\,T_{\mathrm{U}}}{m_{\mathrm{p}}} \right) ^2 \right] . \end{aligned}$$

In this way the heuristic estimate (52) is even closer to the field theoretic calculation in Eq. (51).

It should be noted also that the deformation of the algebra (43) should also affect the Hamiltonian. Therefore, the Rindler frequency $\varOmega$ in Eq. (49) should in principle be modified accordingly. In the present analysis, however, since we consider only small deformations of the quantization rules, we have reasonably neglected those corrections, thus approximating the modified Rindler Hamiltonian with the original one.

Concluding, for small deviations from the canonical quantization, we have found that the Unruh distribution maintains its original thermal spectrum, provided that a new temperature *T* is defined as in Eq. (50).

## Conclusions

In the context of the Generalized Uncertainty Principle, we have computed the correction induced on the Unruh temperature by a deformed fundamental commutator. This result has been obtained by following two independent paths. First, we proceeded in a heuristic way, using very general and reasonable physical considerations. Already at this stage however we have been able to point out a dependence of the deformed Unruh temperature on the cubic power of the acceleration. These considerations have been substantiated and confirmed by means of a full-fledged Quantum Field Theory calculation. This has been achieved by taking into account modified commutation relations for the ladder operators compatible with the GUP in Eq. (5). In the limit of a small deformation of the commutator, we obtained again a dependence of the first correction term on the third power of the acceleration. Besides, the more refined formalism of QFT has helped us to point out an explicit dependence of the deformed Unruh temperature on the Rindler frequency $\varOmega$, which, on the other hand, was reasonably expected. A simple and effective thermodynamic argument has then been used to identify the values of the Rindler frequency $\varOmega$ corresponding to the most probable emission. As a consequence, the QFT calculation is seen to match the heuristic estimate, indeed with almost the same numerical coefficients.

Of course, many avenues for further investigations appear now in front of us. On a technical side, for example, one would like to check whether the QFT corrections to the Unruh temperature are left unchanged if the algebra is deformed at the level of field rather than ladder operators. Further light should be thrown on the relation between the deviation from thermality of the Unruh radiation discussed in this paper, and those found in different contexts (e.g. Refs. [47, 48]). It could also be interesting to extend our formalism to the Hawking effect, for which a heuristic derivation of the temperature from HUP has already been performed in Ref. [35].

Finally, two examples (among the many possible) of applications that could be affected by the results of this paper on a modified Unruh temperature are, broadly speaking: the field of relativistic quantum information theory (e.g. entanglement degradation, entanglement satellite experiments [55, 56]); the corrections induced on analogue gravity experiments (e.g. analogue Unruh radiation in fluids, in BEC, etc. [57, 58]). Much work is still in progress along these directions.

## Footnotes

1 We shall always work with $c=1$, but explicitly show the Newton constant $G_{\mathrm{N}}$ and the Planck constant $\hbar$. The Planck length is defined as $\ell _{\mathrm{p}}=\sqrt{G_{\mathrm{N}}\,\hbar /c^3}\simeq 10^{-35}$ m, the Planck energy as $\mathcal {E}_{\mathrm{p}}\,\ell _{\mathrm{p}}= \hbar \, c/2$, and the Planck mass as $m_{\mathrm{p}} = \mathcal {E}_{\mathrm{p}}/c^2\simeq 10^{-8}$ kg, so that $\ell _{\mathrm{p}}=2G_{\mathrm{N}}\,m_{\mathrm{p}}$ and $2\,\ell _{\mathrm{p}}\,m_{\mathrm{p}}= \hbar$. The Boltzmann constant $k_{\mathrm{B}}$ will be shown explicitly, unless otherwise stated.

2 Note that, although the plane-wave field expansion in Eq. (18) applies to the whole of the Minkowski space-time, the representation (23) in terms of boost-modes does hold only in the Rindler manifold $x>|t|\,\cup \, x<-|t|$ (see Fig. 1). A globally well-defined expansion can be obtained by analytically continuing the modes (21) across the null asymptotes $x=\pm \, t$ (see Ref. [52]). For our purposes, however, it is enough to consider the definition (21) of boost modes.

3 In what follows, the set of Rindler coordinates $\{\eta , \xi \}$ will be also denoted by $\mathbf{x}$; therefore, such a symbol will refer to a space-time point, rather than its representative in a particular coordinate system.

4 Using $c\ne 1$ for sake of clarity, the proper frequency $\omega$ measured by a Rindler observer is obtained from $\omega \,\tau = \omega (\eta c/a)=(\omega c/a)\eta \equiv \varOmega \,\eta$.
